# miR-135b Contributes to the Radioresistance by Targeting GSK3β in Human Glioblastoma Multiforme Cells

**DOI:** 10.1371/journal.pone.0108810

**Published:** 2014-09-29

**Authors:** Songhua Xiao, Zhen Yang, Ruiyan Lv, Jia Zhao, Ming Wu, Yiwei Liao, Qing Liu

**Affiliations:** 1 Department of Neurology, Sun Yat-sen Memorial Hospital, Sun Yat-sen University, Guanzhou, Guangdong, People's Republic of China; 2 Department of Hypertension and Vascular Disease, The First Affiliated Hospital, Sun Yat-Sen University, Guanzhou, Guangdong, People's Republic of China; 3 Department of Neurosurgery, Xiangya Hospital, Central South university, Changsha, Hunan, People's Republic of China; University Hospital of Navarra, Spain

## Abstract

Radioresistance remains a major challenge in the treatment of glioblastoma multiforme (GBM). Recent data strongly suggests the important role of miRNAs in cancer progression and therapeutic response. Here, we have established a radioresistant human GBM cell line U87R derived from parental U87 and found miR-135b expression was upregulated in U87R cells. miR-135b knockdown reversed radioresistance of U87R cells, and miR-135b overexpression enhanced radioresistance of U87 cells. Mechanically, bioinformatics analysis combined with experimental analysis demonstrated GSK3β (Glycogen synthase kinase 3 beta) was a novel direct target of miR-135b. Moreover, GSK3β protein expression was downregulated in U87R cells and restored expression of GSK3β increased radiosensitivity of U87R cells. In addition, clinical data indicated that the expression of miR-135b or GSK3β was significantly association with IR resistance of GBM samples. Our findings suggest miR-135b is involved in the radioresistance of human GBM cells and miR-135b-GSK3β axis may be a novel candidate for developing rational therapeutic strategies for human GBM treatment.

## Introduction

Gliomas are the most common type of primary brain tumors in adults and persist as serious clinical and scientific problems [1]. Survival depends heavily on the histological grade of the tumor, but patients afflicted with the most malignant glioma, glioblastoma multiforme (GBM). survive on average about 15 months. Despite advances in current multi-modal treatment options, the overall prognosis of patients with GBM remains dismal [2]. These include rapidness and invasiveness of tumor growth, the genetic heterogeneity of the tumors, and our poor understanding of the molecular mechanisms governing disease manifestation and progression [3], [4]. Ionizing radiation (IR) plays a major role in the treatment of patients with GBM. Factually, the efficacy of this therapeutic modality is often limited by the occurrence of radioresistance [5]. However, the molecular mechanisms responsible for the radioresistance of human GBM are still not clear yet.

Recently, it has been recognized that a class of endogenous, small, non-protein coding single-stranded RNA molecules, termed microRNA (miRNA), plays a crucial role in the post-transcriptional regulation of gene expression. More and more reports have demonstrated that miRNAs are aberrantly expressed in many human cancers, functions as oncogenes and tumor suppressors [6]. Some miRNAs have been demonstrated perhaps to play key roles in tumorigenesis, progression, invasion or metastasis in human GBM, such as miR-181, miR-200b, miR-182, miR-381, miR-142-3p and others [4], [7]–[9]. However, the role of miRNAs in radioresistance of human GBM largely remains unknown. In the present study, compared to its parental cell line U87, we show miR-135b is upregulated in radioresistant human GBM cell line U87R, which targets Glycogen synthase kinase 3 beta (GSK3β). Our findings suggest that miR-135b and GSK3β are potential biomarkers to estimate the sensitivity of human GBM to radiotherapy and help to developing rational therapeutic strategies.

## Materials and Methods

### Tissue specimens

We obtained frozen tissue samples of 30 human GBM tissues and 30 normal brain (NB) tissues from the Xiangya Hospital of the Central South University, Hunan, China between March 2008 and November 2010. The study was approved by the Ethical Committee of the Faculty of Medicine, the Central South University, and written informed consent was obtained from every subject. The collection and use of tissues followed the procedures that are in accordance with the ethical standards as formulated in the Helsinki. Tumor samples were diagnosed by 2 pathologists who were blinded to patient data using the World Health Organization (WHO) system. Clinical data, including gender, age, follow-up, and outcome, were obtained from the medical records.

### Cell culture

Human GBM cell line U87 and its radioresistant derivate cell line U87R were cultured in DMEM (Life Technologies) supplemented with 10% fetal bovine serum (Life Technologies) in a humidified cell incubator with an atmosphere of CO2 at 37°C. Exponentially growing cells were used for experiments.

### Survival foci formation assay

Cells in exponential growth phase were plated into a six-well plate at 2000 cells/well and treated with a range of radiation doses (0, 2 and 4 Gy) after adhesion. When most cell clones had reached >50 cells, they were stained with 0.06% crystal violet, and foci number was counted.

### Cell proliferation assay

Cell proliferation was monitored by the MTS assay using the CellTiter96AQueous One Solution Cell Proliferation Assay kit (Promega) according to the manufacturer's instructions. Cells were seeded into 96-well plates at 2000 cells/well (0.20 ml/well), and irradiated with 2 Gy or not. The cell proliferation assay was performed on days 0, 1, 2, 3 and 4 by incubation with MTS (0.02 ml/well). After 2 h further incubation, the absorbance at 490 nm of each well was recorded on the BiotexELX800 and the absorbance represented the cell number.

### Quantitative RT-PCR analysis (qRT-PCR)

Total RNAs were extracted from cells with TRIzol reagent (Invitrogen). For the detection of GSK3β mRNA, cDNA was synthesized from 1 µg of total RNA by means of the reverse reaction kit according, which was used in accordance with the manufacturer's instructions (Promega). Human GAPDH was amplified in parallel as an internal control. The primers were: GAPDH was used as an internal control and the qRT-PCR was repeated three times. The primers for GAPDH were: forward primer 5′-ATTCCATGGCACCGTCAAGGCTGA-3′, reverse primer 5′-TTCTCCATGGTGGTGAAGACGCCA-3′; for GSK3β were: forward primer 5′- GACTAAGGTCTTCCGACCCC-3′, reverse primer 5′- TTAGCATCTGACGCTGCTGT-3′.

### CellsTransfection

miR-135b mimics, miR-135b inhibitor and relative controls were purchased from Ambion. Cells were trypsinised, counted and seeded onto 6-well plates the day before transfection to ensure 70% cell confluence on the day of transfection. The transfection of mimics, inhibitor, pLV-GFP-GSK3β vector and related controls was carried out using Lipofectamine 2000 (Invitrogen) in accordance with the manufacturer's procedure. The mimics, inhibitor and controls were used at a final concentration of 100 nM. At 48 h post-transfection, follow-up experiments were performed.

### Construction of luc-UTR vectors

The full-length GSK3β 3′-UTR was cloned into the EcoRI and HindIII sites of the pMIR-REPORT luciferase vector (Ambion, Austin, TX, U.S.) using PCR generated fragment. A Luc-mut vector in which the first seven nucleotides complementary to the miR-135b seed-region were mutated by site-directed mutagenesis (Stratagene) served as a mutant control.

### Luciferase assay

Luc-wt, Luc-mut, and Luc-ctrl were co-transfected within vitro-produced miR-135b into U87 cells. The pMIR-REPORT β-galactosidase control vector was transfected and served as a control. Luciferase activity was measured in cell lysates 48 h after transfection using a dual-light luminescent reporter gene assay kit (Applied Biosystems).Results were normalized against β-galactosidase activity.

### Western blot

Cell protein lysates, cytosol protein or nuclear protein was separated in 10% SDS-polyacrylamide gels, electrophoretically transferred to polyvinylidene difluoride membranes (Millipore), then detected with mouse monoclonal antibody for RAB21 (sc-81917), mouse monoclonal antibody for GSK3β and LZTS1 (Santa Cruz), mouse monoclonal antibody for β-actin (Abcam) and commercial ECL kit (Pierce). The intensity of protein fragments was quantified using ChemicalDocTM XRS+ (Bio-Rad).

### Immunofluorescence assay

For immunofluorescence assay cells were seeded on coverslips, fixed with 3% paraformaldehyde for 20 min and then permeabilized with 0.1% Triton X-100. Cells were incubated with antibodies against GSK3β for 2 hours at room temperature. Then, cells were incubated with species-specific Alexa488-conjugated secondary antibodies (1∶2000; Invitrogen) for 1 h at room temperature. Nuclei were counterstained with 4′,6-diamidino-2-phenylindole (DAPI, 1 µg/mL).

### Statistical analysis

Quantitative results were expressed as the mean ± standard deviation. Statistical analysis was carried out with Statistical Package for Social Science (SPSS for Windows Version 16.0. USA). Student t-test was used to evaluate the statistical significance. A *p* value <0.05 or 0.01 was set as the criteria for statistical significance.

## Results

### Biological characteristics of U87R cells

**Figure 1:**
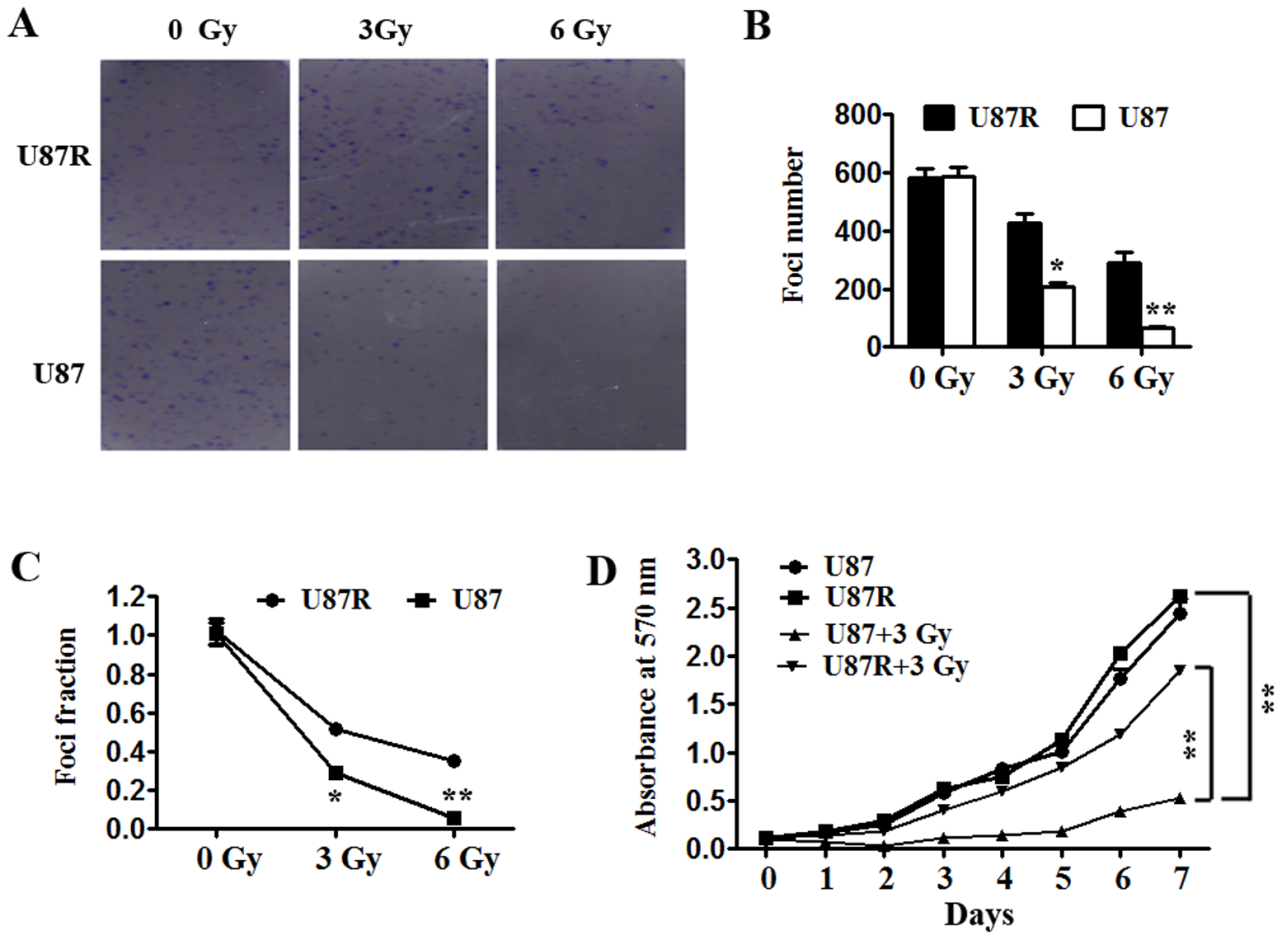
U87R cell line is radioresistant. (A, B) U87R cells has more foci number than U87 cells. Indicated cells were exposed to several radiation dose and the experiments were repeated three times. The numbers of foci formation were presented as bar graphs. *p<0.05, **p<0.01. (C) U87R cells have more foci fraction than U87 cells. Foci fractions were calculated by dividing the number of colonies formed after radiation by the corresponding number of colonies formed without radiation from experiments in (A and B). (D) U87R cells get less effect than U87 cells from radiation on growth pattern. U87R and U87 cells plated in 96-well culture plates were exposed to radiation with 3 Gy cell growth was monitored by measuring cell numbers with MTS assay. **p<0.01.

In order to explore the mechanism responsible for radioresistance in human GBM, firstly, we established aradioresistant human GBM cell line. To generate a radioresistant human GBM cell line U87R, we exposed U87 cells to a range of doses of radiation (1, 2, 4, 6 and 8 Gy) over a period of 12 months [10], [11]. Cells were lonizing radiation (IR) at least three times at each dose and cells did not receive next radiation until they got an 80% confluence after each radiation. To verify the radioresistant phenotype, we radiated U87 and U87R cells and examined them by survival foci formation assay. U87 and U87R cells were irradiated with 0, 3 and 6 Gy and examined by survival foci formation assay. Compared to U87, U87R showed no change of foci formation ability when radiation was absent, but gained more foci formation and higher survival fractions when exposed to radiation (Figure 1A–C). The effect of radiation on cell growth was also examined by subjecting U87 and U87R cells to 3 Gy IR. As shown in Figure 1D, the U87R cell line had more cell numbers than U87 after radiation. So that, U87R cells is more radioresistant than parental U87 cells.

### miR-135b enhances radioresistance of U87 cells

**Figure 2:**
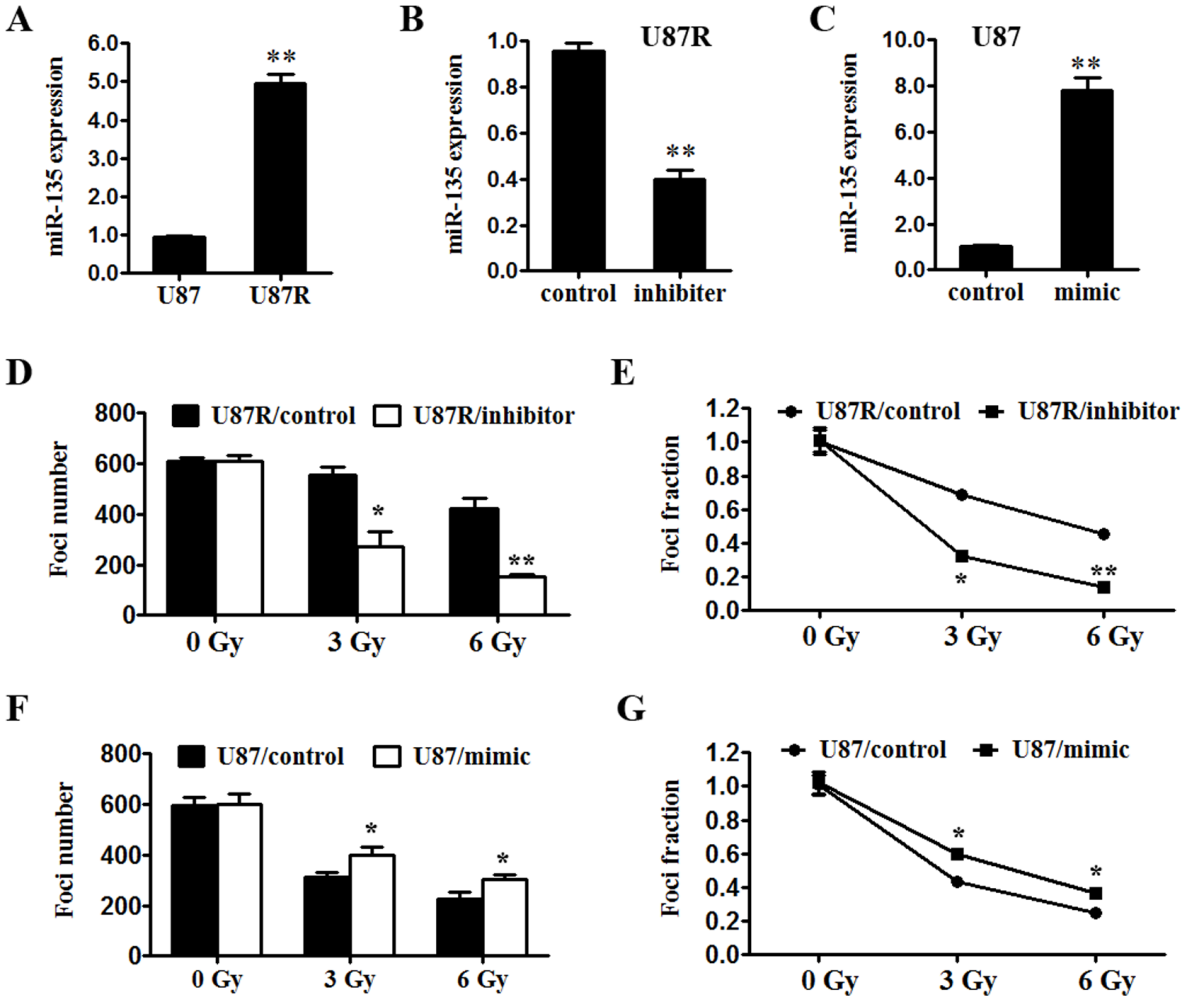
miR-135b contributes to radioresistance in U87 cells. (A) miR-135b expression increases in U87R cells, compared to U87 cells. (B and C) miR-135b expression was modulated using miR-135b inhibitor or mimics respectively. (D–E) function inhibition of miR-135b reversed radioresistance of U87R cells detected by foci formation assay. (F–G) ectopic expression of miR-135b enhanced radioresistance of U87 cells detected by foci formation assay. *p<0.05.

Using miRNA specific RT-qPCR, we found miR-135b expression was about 5.17 fold higher in U87R cells than that in U87 cells (Figure 2A). To investigate whether miR-135b has a direct function in modulating the radioresistance in U87 cells, we used loss-of-function and gain-of-function approaches in U87R and U87 cells, respectively. So we inhibited miR-135b function with miR-135b specific inhibitor in U87R cells to further characterize its biological function (Figure 2B). qRT-PCR and Western blot [12] analysis showed that the transfection was successful (Figure S1). miR-135b inhibition significantly increased the radiosensitivity of U87R cells measured by survival foci formation assay (Figure S2 and Figure 2D–E). Conversely, the ectopic expression of miR-135b in U87 cells was achieved by miR-135b mimics transfection (Figure 2C). Overexpression of miR-135b with mimics in U87 cells efficiently increased the radioresistance measured by survival foci formation assay (Figure 2F–G).

### miR-135b directly targets GSK3β

**Figure 3:**
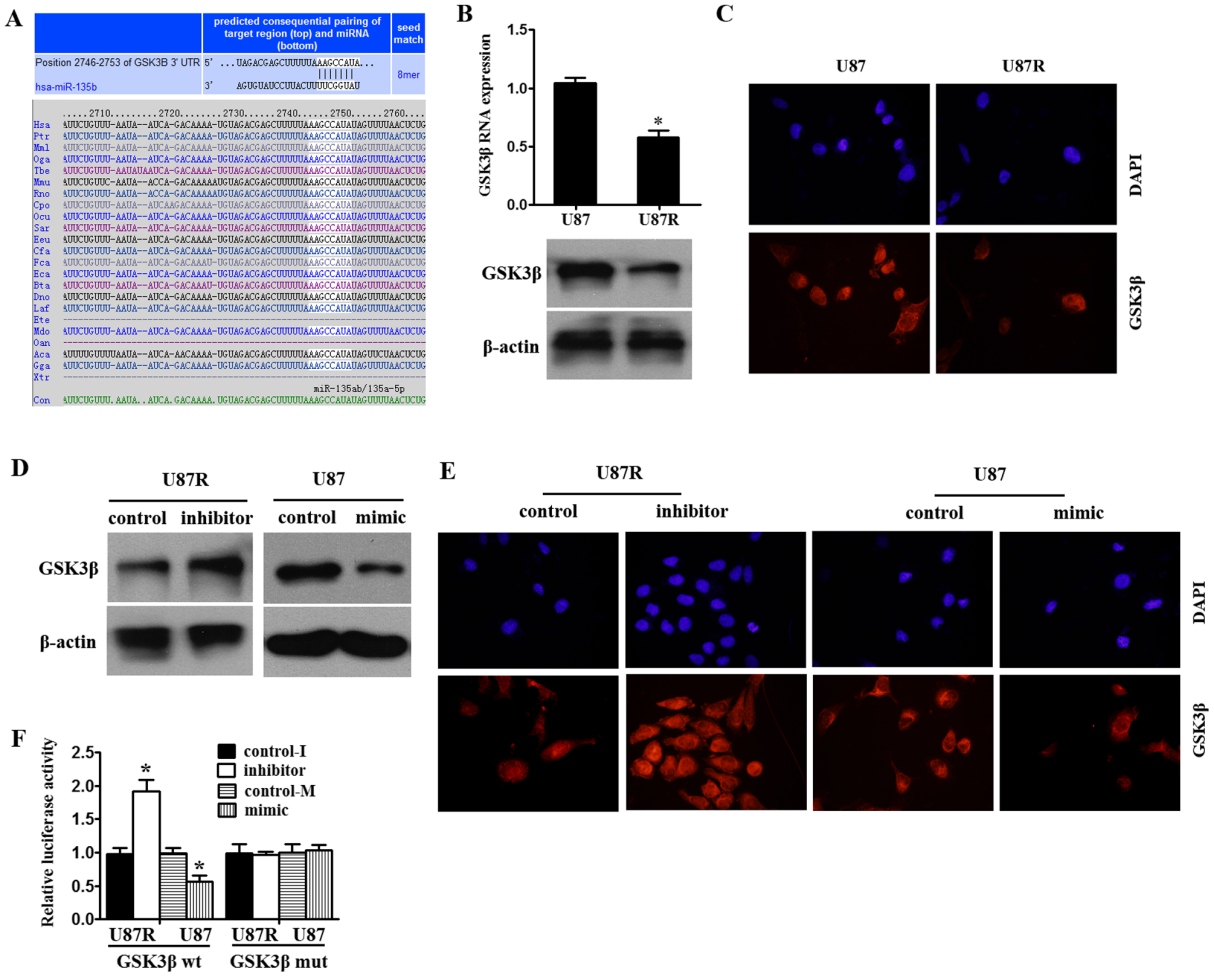
miR-135b targets GSK3β. (A) Schematic of predicted miR-135b site in the 3′UTR of GSK3β mRNA, which broadly conserved among vertebrates. (B) GSK3β expression was much lower in U87R cells compared to U87 cells at protein level and mRNA level by qRT-PCR and Western blot. (C) GSK3β expression was much lower in U87R cells compared to U87 cells at protein level by immunofluorescence. (D) miR-135b expression interference negatively regulated GSK3β protein expression by Western blot. (E) miR-135b expression interference negatively regulated GSK3β protein expression by immunofluorescence. (F) Luciferase reporter assays were performed after transfection with indicated pMIR-Report plasmids and a renilla transfection control plasmid, co-transfected with miR-135, or relevant scramble controls. Data shown were the mean±SD of six replicates and were representative of three independent experiments. *p<0.05.

To analyze the molecular mechanisms of miR-135b involvement in radioresistance of human GBM cells, online softwares TargetScan was used to search for potential miR-135b target genes. A predict potential target of miR-135b, and GSK3β with critically conserved binding site was selected for further molecular and functional confirmation (Figure 3A). To investigate the co-relation between miR-135b and GSK3β, the expression of GSK3β at mRNA and protein levels were detected in U87 and U87R cells. The expression of GSK3β and mRNA protein in U87R cells was much lower than that in U87 cells (Figure 3B, C). Moreover, GSK3β protein level in U87R cells significantly increased after miR-135b inhibitor transfection. Conversely, GSK3β protein level in U87 cells decreased after miR-135b mimics transfection (Figure 3D, E). To assess whether GSK3β is a direct target of miR-135b, we subcloned the full-length 3′-UTR of GSK3β into the luciferase reporter vector. It's shown that addition of in vitro-produced miR-135b suppressed the luciferase activity of the 3′-UTR of GSK3β upon co-transfection of the luciferase vector (wild-type, mutant) with the in vitro-produced microRNAs (miR-135b mimic or scramble control) into U87 cells and vitro-produced microRNAs (miR-135b inhibitor or scramble control) into U87R cells (Figure 3F). This inhibition was abolished when the seed sequences of the miR-135b were mutated in the Luc-mut vector. These results strongly demonstrated the specificity of miR-135 targeting GSK3β.

### GSK3β overexpression reverses the radio-resistance of U87R cells

**Figure 4:**
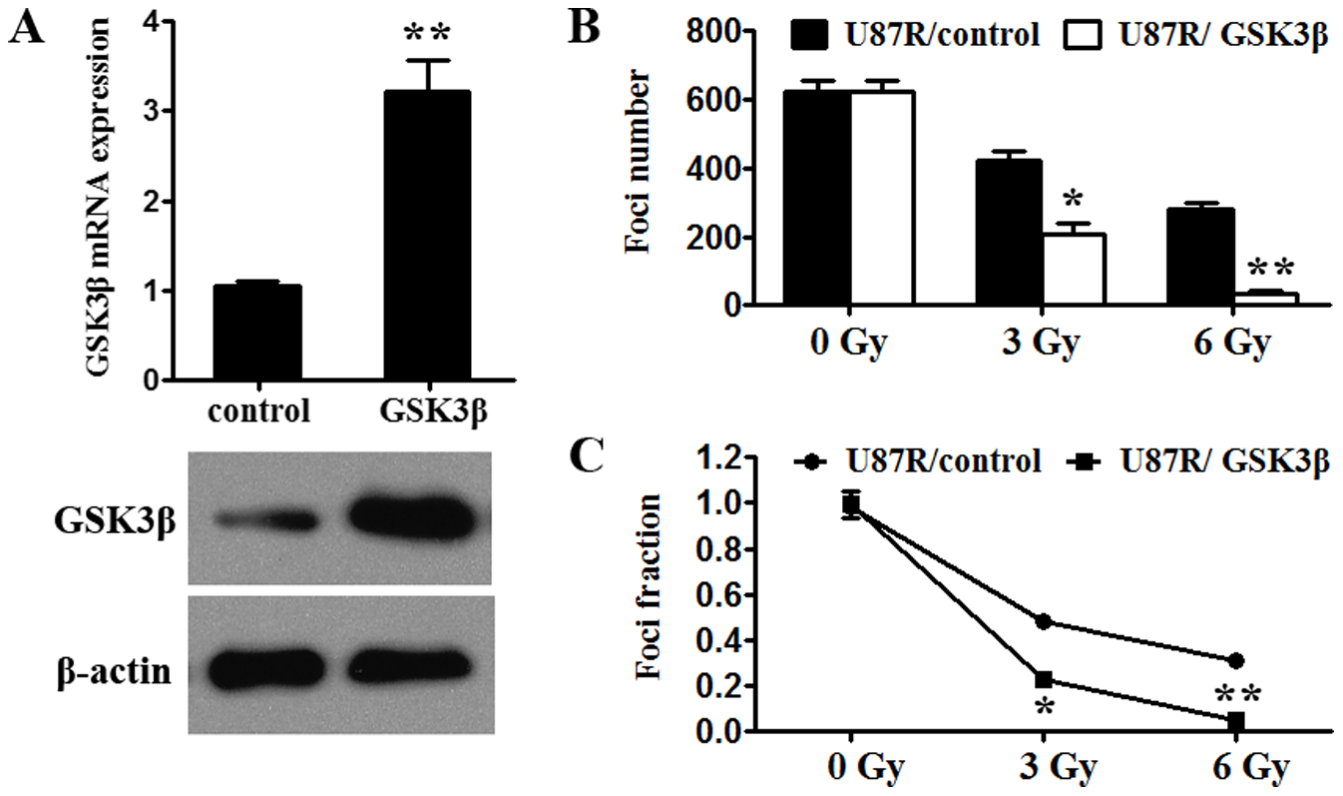
GSK3β overexpression reverses radioresistance of U87R cells. (A) Transfection of GSK3β vector increased GSK3β protein expression. (B–C) GSK3β ovexpression reversed radioresistance of U87R cells detected by foci formation assay. *p<0.05.

GSK3β is a direct target of miR-135b in U87R cells. To investigate the role of GSK3β in human GBM radiosensitivity, GSK3β overexpression with pLV-GFP-GSK3β vector was applied. pLV-GFP-GSK3β vector transfection significantly increased GSK3β protein level (Figure 4A). Then, we observed the functional effect of restored GSK3β expression on radiosensitivity and found GSK3β overexpression significantly reversed radioresistance of U87R cells detected by survival foci formation assay (Figure 4B–C).

### The association of miR-135b or GSK3β expression with IR resistance is clinically relevant

**Figure 5:**
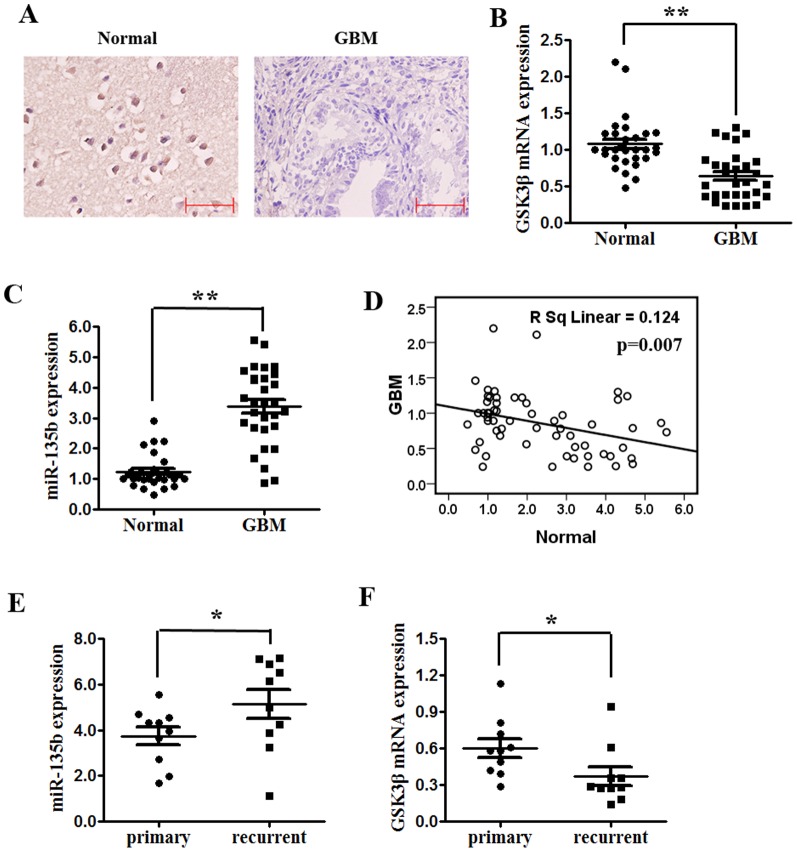
The association of miR-135b or GSK3β expression with IR resistance is clinically relevant. (A) The GSK3β protein levels of were detected in a cohort of 30 GBM tissues and 30 normal brain tissues using immunohistochemistry assays. (B) Quantitation of GSK3β expression in 30 glioblastoma multiforme (GBM) samples and 30 normal brain tissues (Normal). GAPDH served as the loading control. Alteration of expression is shown as box plot presentations. **p<0.01. (C) Quantitation of miR-135b or GSK3β expression in 30 glioblastoma multiforme (GBM) samples and 30 normal brain tissues (Normal). GAPDH served as the loading control. Alteration of expression is shown as box plot presentations. **p<0.01. (D) The correlation analysis showed that the expression of miR-135b was negatively correlated with the expression of GSK3β proteins in normal brain and GBM tissues (p = 0.07). (E, F)The relative expression level of miR-135b or GSK3β was determined by qRT-PCR in ten paired samples of primary and recurrent GBM. *p<0.05.

To determine whether the association of miR-135b or GSK3β expression with IR resistance is clinically relevant, we examined the expression levels of miR-135b or GSK3β in a cohort of 30 GBM tissues and 30 normal brain tissues using qRT-PCR (miR-135b and GSK3β) and immunohistochemistry (GSK3β) assays. Our result showed that GSK3β protein in approximate 83.3% (25/30) samples was positive among the 30 normal brain tissues. However there was approximately 13.3% (4/30) positivity in 30 GBM tissues. The representative images are in Figure 5A. GSK3β mRNA was found to be significantly downregulated in these GBM specimens compared with normal brain tissues (p<0.01, Figure 5B). But miR-135b was found to be significantly upregulated in these GBM specimens compared with normal brain tissues (p<0.01, Figure 5C). The correlation analysis showed that the expression of miR-135b was negatively correlated with the expression of GSK3β proteins in normal brain and GBM tissues (p = 0.07, Figure 5D). Furthermore, we examined the miR-135b or mRNA expression of GSK3β in tumor tissues from patients who received IR treatment. miR-135b or GSK3β was measured in ten pairs of frozen GBM specimens, including primary tumors and the corresponding recurrent tumors. qRT-PCR data showed that miR-135b was dramatically elevated in the recurrent tumors compared to the primary tumors after IR treatment, but the mRNA expression of GSK3β was dramatically declined in the recurrent tumors compared to the primary tumors after IR treatment (p<0.01, Figure 5E, F), indicating that the glioma cells with high expression of miR-135b or GSK3β were more resistant to the IR and more susceptible to death, whereas the glioma cells with low miR-135b or GSK3β levels were more sensitive to IR. Together, these results suggest that miR-135b or GSK3β is of clinical significance as a mediator of IR resistance.

## Discussion

Intrinsic and acquired resistance of therapeutic-resistance including chemoresistance and radioresistance is the major clinical obstacle in cancer treatment. Although mechanisms for therapeutic-resistance have been extensively investigated using established chemo-or-radio-resistant cell models [11], [13], [14], but they are still not fully understood. Recently, microRNAs are regulators of central cellular processes and are implicated in the pathogenesis and prognosis of human cancers. The importance of miRNAs as potential prognostic indicators or therapeutic targets for cancers are underscored by their functions in regulating fundamental cellular processes, such as cell proliferation, differentiation, invasion and apoptosis [6], [15]. MicroRNAs also modulate responses to anti-cancer therapy. In the context of radiation oncology microRNAs were found to modulate cell death and proliferation after irradiation [11]. For example, miR-1285 was observed in the present study might possibly lead to increased radioresistance in subsequent radiotherapy sessions. Furthermore, irradiation-induced changes in microRNA expression levels might also affect migration and metastasis of surviving cells [11]. IR-induced over-expression of miR-151-5p might enhance dissemination and migration of malignant cells during a course of radiation therapy, since miR-151-5p was found to increase migration and intra-hepatic metastasis in hepatocellular carcinoma [16]. Another candidate for regulating responsiveness to anticancer therapy is the let-7 family, although certain members of the let-7 family had different effects on radiation sensitivity in A549 lung cancer cells [17]. let-7a overexpression was shown to increase radiation sensitivity in lung cancer cells [18].

In regard to human GBM, ionizing radiation (IR) is a key component of standard therapy for GBM patients. However, acquired resistance limits the therapeutic efficacy of IR [5]. Therefore, the identification of targets responsible for IR resistance is critical for successful GBM treatment. In this study, we show, for the first time, that miR-135b functions as a novel mediator of IR resistance in glioma cells. In order to explore the mechanisms responsible for radioresistance of human GBM, we established a radioresistant human GBM cell line U87R and found miR-135b was upregulated in U87R cells. Expectedly, miR-135b knockdown significantly increased the radiosensitivity of U87R cells. However, miR-135b overexpression obviously enhanced the radioresistance of U87 cells. Previous evidence has indicated that miR-135b is overexpressed in lung [12], colon [19], [20], breast [21] and prostate [22] cancers, which strongly suggests a general role in different types of cancers. The ability of miR-135b to target multiple tumour suppressors indicates an indiscriminate ability to promote tumour progression and metastasis. Other researchers have found that miR-135b may also contribute to mediate NPM-ALKassociated oncogenicity in large-cell lymphomas [23]. A miR-135b antagomir (Antago-135), which is able to functionally suppress miR-135b, effectively reduced metastasis and tumour burden, which suggests the potential for the development of miR-135b antagonists for lung cancer therapy [12]. Here, we firstly demonstrate miR-135b is involved in the radioresistance in human GBM cells.

As the mechanism responsible of miR-135b in human GBM cells, we found miR-135b directly targets GSK3β whose mRNA and protein expression is downregulated in U87R cells with endogenous upregulation of miR-135b expression. GSK3β is a serine/threonine protein kinase involved in glycogen metabolism and the Wnt signaling pathway, which plays important roles in embryonic development pathway and tumourigenesis. Active GSK3β is able to phosphorylate substrates, such as β-catenin and Tau, resulting in ubiquitin-mediated degradation [24]. Wnt signaling inactivates GSK3β through the phosphorylation of Ser9 residue and prevents it from phosphorylating β-catenin, thus stabilizing GSK3β in the cytoplasm [25]. miR-135b has also been reported to induce Wnt signaling pathway by the suppression of APC in colorectal cancers [26] and lung cancer [12]. Studies have shown clear regulatory mechanisms of GSK3β activity in various cancer, however, that of GSK3β expression remains unclear in GBM. Here, we demonstrated miR-135b could direct suppress GSK3β expression at mRNA and protein level in human GBM cells. In addition, we examined the expression levels of miR-135b and GSK3β in human GBM specimens and found that miR-135b was significantly upregulated in primary GBM tissues. On the contrary, GSK3β was significantly downregulated in primary GBM tissues. More importantly, we found that elevated expression of miR-135b and GSK3β are associated with recurrent GBM patients who underwent IR therapy. To the best of our knowledge, we will explore the detailed mechanism responsible for miR-135b-GSK3β axis in modulating radiosensitivity in human GBM cells.

In conclusion, the U87R and U87 cell lines are useful models for clarifying the radioresistant mechanisms in human GBM. miR-135b is upregulated in U87R cells and contributes radioresistance in U87 cells. As for mechanism, we found GSK3β is a direct target of miR-135b, and ectopic GSK3β expression significantly reverses radioresistance in U87R cells. Thus, our findings suggest miR-135b-GSK3β axis will be valuable biomarkers for the radiosensitivity and related interference will be worthy therapeutic strategies for human GBM patients.

## Supporting Information

Figure S1 **qRT-PCR measured the miR-135 expression levels in U87 cells transfected with miR-135b mimics as well as U87R cells transfected with miR-26a inhibitors.** Western blot measured the LZTS1 protein (a target gene of miR-135b) expression levels in U87 cells transfected with miR-135b mimics.
(TIF)

Figure S2 **Micro-photographs after 48 h of transfection with miR-135b inhibitor and the corresponding controls.**
(TIF)
