# Supplementary figures and images for: miR-135b Contributes to the Radioresistance by Targeting GSK3β in Human Glioblastoma Multiforme Cells
Source: PLoS One. 2014 Sep 29;9(9):e108810. doi: 10.1371/journal.pone.0108810 (PMC4181861; doi:10.1371/journal.pone.0108810)

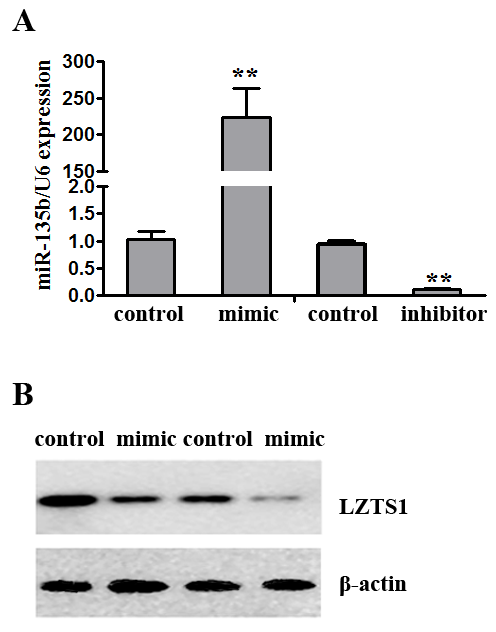

Supplement: Figure S1 — qRT-PCR measured the miR-135 expression levels in U87 cells transfected with miR-135b mimics as well as U87R cells transfected with miR-26a inhibitors. Western blot measured the LZTS1 protein (a target gene of miR-135b) expression levels in U87 cells transfected with miR-135b mimics. (TIF) [file pone.0108810.s001.tif]

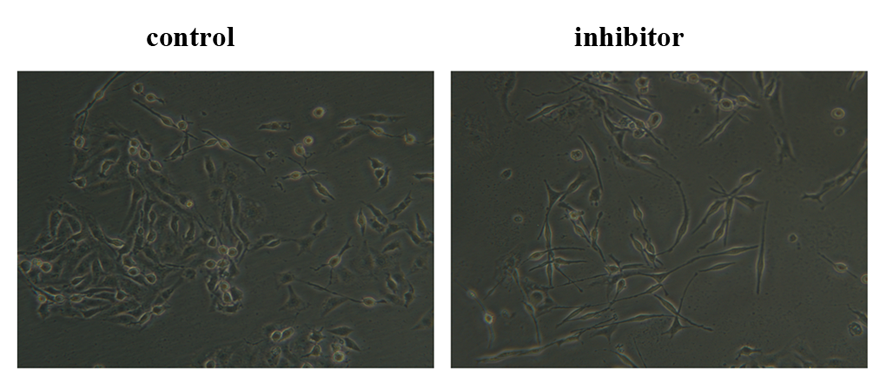

Supplement: Figure S2 — Micro-photographs after 48 h of transfection with miR-135b inhibitor and the corresponding controls. (TIF) [file pone.0108810.s002.tif]
